# 

**Published:** 1875-11

**Authors:** 


### Editorial and Miscellaneous.

*To our Delinquent Subscribers.*

Simultaneously with the mailing of the August number of this journal, we furnished each delinquent subscriber a statement of his account up to the first of January, 1876, requesting his early attention to the matter. Some of these have responded, while many have, up to the present, failed to notice our request. It is certainly absurd to presume that any physician in the regular practice is unable to raise the small sum of three dollars in thirty days, and we will not indulge the suspicion that any of the gentlemen now in arrears for subscripton are so destitute of honor as to intentionally avoid the payment due. There is, therefore, but one key with which to solve the problem of their failure to respond, and that is the fact of negligence which so cruelly afflicts many good men, or a forgetfulness that while a little more excusable is none the less an evil to us.

The preparation of the journal for the profession, if saved from these delays and losses, would indeed be a pleasure, but we cannot afford the time required to manage its business or financial details, unless promptness should characterize our subscribers. We, therefore, make a personal appeal to each of these, and request him no longer to neglect or forget the obligation which he has assumed, but to wake up and act at once in remitting his dues. If any should treat this appeal with indifference, we shall be compelled, and pledge our honor, to send his account for collection through such a channel as will incur additional expense. This is business; we mean what we say.

### COLLABORATORS AND CONTRIBUTORS.

**GEORGIA.**

James A. Long, M.D.
W. L. M. Harris, M.D.
H. L Battle, M.D.
W. C. Musgrove, M D
L B. Bouchelle, M.D.
Thomas Burdell, M.D.
E. H. W. Hunter, M.D.
V. S Hitt, M.D.
J. E. Pope, M.D.
J. C. Wisdom, M.D.
W. W. Leake, M.D.
S. G. White, M.D.
W. H. Hall, M.D.
G. D Case, M.D.
W. T. Grant, M.D.
J. M. Tules, M.D.
J. N. Cheney, M.D.
T. M. Shaw, M.D.
T. S. Mitchell, M.D.
H. B. Lee, M.D.
C. B. Nottingham, M.D.
W. L. Kirkpatrick, M.D.

**KENTUCKY.**

W. S. Taylor, M.D.
B. W. Stone, M.D.
Charles Kearns, M.D.

**TEXAS.**

S. M. Welch, M.D.
T. J. Heard, M.D.
L. R. Lincecum, M.D.

**TENNESSEE.**

J. D. Tucker, M.D.

F. A. Ramsey, M.D.

J. W. Hayne, M.D.

F. K. Bailey, M.D.

**WEST VIRGINIA.**

N. D. Tobey, M.D.

**ALABAMA.**

J. C. Knox, M.D.
Geo. F. Taylor, M.D.
J. B. Barnett, M.D.
S. M. Hogan, M.D.
J. T. Searcy, M.D.
A. C. Edwards, M.D.
M. W. Morton, M.D.
J. D. Rush, M.D.
J. J. Grace, M.D.

**NORTH CAROLINA.**

J. B. Hughes, M.D.
S. S. Satchwell, M.D.
A. B. Pierce, M.D.
H. Kelly, M.D.
Geo. A. Foote, M.D.
E. A. Anderson, M.D
H. T. Bahnson, M.D.
John W. Booth, M.D.
R. S. Hallsey, M.D.

**SOUTH CAROLINA.**

E. T. McSwain, M.D.
G. M. Rivers, M.D.

**OHIO.**

J. Q. A. Hudson, M.D
C. H. Tidd, M.D.
J. C. Bishop, M.D.
W. P. Wells, M.D.

**NEW YORK.**

Ely Van DeWarker, M. D.
C. C. F. Gay, M.D.
J. S. Bailey, M.D.

**COLORADO.**

Wm. R. Whitehead, M.D.

**VIRGINIA.**

F. Horner, Jr., M.D.
R. J. Preston, M.D.
J. S. Wharton, M.D.
Sam'l P. Ripley, M.D.
E. A. Drewry, M.D.
Rob B. Tunstall, M.D.
W. F. Barr, M.D.
L. B. Anderson, M.D.
J. N. Upshur, M.D.
B. P. Reese, M.D.

**MISSISSIPPI.**

C. B. Gallaway, M.D.
Edward Lea, M.D.
S. V. D. Hill, M.D.
E. T. Henry, M.D.
T. P. Lockwood, M.D.
Robert Kells, M.D.
W. L. Lipscomb, M.D.
W. T. Beall, M.D.
A. G. Smythe, M.D.
J. M. Lewis, M.D.
J. H. Payne, M.D.
T. H. Mayo, M.D.
E. Cutter, M.D.

**ARKANSAS.**

A. D. Hodge, M.D.
J. K. Hodge, M.D.
A. W. Hobson, M.D.
C. D. Hodge, M.D.

**INDIANA.**

G. A. Dyer, M.D.
A. Patton, M.D.
Robert Robson, M.D.

**ILLINOIS.**

John H. Weir, M.D.

**OREGON.**

W. M. Cusick, M.D.

*To the Subscribers of the Southern Medical Record:*

The necessity for the existence in the South of a monthly magazine such as the SOUTHERN MEDICAL RECORD, is unquestionable. Devoted, as it is, to the dissemination of all that is worthiest and most valuable in medical science, it has met the unqualified approval of the Medical Profession throughout the South; and as regards the good opinion of its readers, the RECORD can, in truth, be designated as a splendid success. The only drawback under which the RECORD, during the present year, has suffered, was in the punctuality of its monthly issues.

To obviate the recurrence of this evil, we propose to place the RECORD for 1876 upon a basis which shall ensure the utmost punctuality in this respect.

In the December number, we shall fully explain the metnod by which we intend to consummate this desirable end. In the meantime we appeal to every subscriber to continue his subscription to the RECORD, and to procure at least one new subscriber to our lists.

As a further inducement, we offer to every subscriber sending in one new name, either a copy of Powell's Pocket Formulary, or he can retain a half dollar of his own subscription price for 1876.

Remittances from all who have forwarded their dues since the September number, will be acknowledged in the December number. We earnestly solicit all who are delinquents in the matter of remittances, to forward their dues at once. No indebtedness for past subscriptions can be allowed, as we desire to begin the new year untrammelled in this respect, and to be in a condition which shall afford opportunities to improve the RECORD and augment its value.

Respectfully,

T. S. POWELL, M. D.

